# Improving Cancer MDT performance in Western Sydney – three years’ experience

**DOI:** 10.1186/s12913-021-06203-y

**Published:** 2021-03-06

**Authors:** Lynleigh Evans, Yiren Liu, Brendan Donovan, Terence Kwan, Karen Byth, Paul Harnett

**Affiliations:** 1 Sydney West – Translational Cancer Research Centre, Western Sydney Local Health District, PO Box 533 Wentworthville, Sydney, NSW 2145 Australia; 2 Tumour Program Strengthening Initiative innovation manager (2019), Western Sydney Local Health District, Sydney, Australia; 3 Tumour Program Strengthening Initiative innovation manager (2018), Western Sydney Local Health District, Sydney, Australia; 4 Faculty of Engineering and IT, University of Sydney, Sydney, Australia; 5 Research and Education Network, Western Sydney Local Health District, Sydney, Australia

**Keywords:** Multidisciplinary team, MDT, Cancer, Quality improvement, Behaviour change

## Abstract

**Background:**

While multidisciplinary teams (MDTs) are now considered an essential part of cancer care decision-making, how they perform varies widely. The authors hypothesised that a comprehensive, multipronged improvement program, and associated annual member survey, could strengthen MDT performance across a whole cancer service.

**Methods:**

The study comprised the introduction of a structured program, the Tumour Program Strengthening Initiative (TPSI) linked with an annual survey of member’s perceptions of their performance. Three iterations of the survey have been completed (2017, 2018 and 2019). Generalised estimating equations (GEEs) were used to test for a difference in the proportion of positive survey responses between 2017 and 2019 adjusted for team clustering.

**Results:**

Twelve teams participated in TPSI. One hundred twenty-nine, 118 and 146 members completed the survey in 2017, 2018 and 2019, respectively. Of the 17 questions that were asked in all three years, nine showed significant improvement and, of these, five were highly significant. Documenting consensus, developing Terms of Reference (TORs), establishing referral criteria and referring to clinical practice guidelines showed most improvement. Questions related to patient considerations, professional development and quality improvement (QI) activities showed no significant change.

**Conclusions:**

TPSI resulted in sustained and significant improvement. The MDT survey not only allowed MDT members to identify their strengths and weaknesses but also provided insights for management to flag priority areas for further support. Overall program improvement reflected the strengthening of the weakest teams as well as further improvement in highly performing MDTs. Importantly, the initiative has the potential to achieve behaviour change amongst clinicians.

**Supplementary Information:**

The online version contains supplementary material available at 10.1186/s12913-021-06203-y.

## Background

Multidisciplinary teams (MDTs) are considered the gold standard of care for patients with cancer [1–7]. They ensure that a diverse range of expert opinions guide management decision-making. A growing body of evidence suggests that MDTs lead to improved diagnostic and treatment recommendations, clinical outcomes, and coordination of care [1–7].

Both how MDTs function and their role in decision-making vary widely [8]. Such variations have been shown to influence the quality of diagnostic and treatment decisions [9, 10]. While previous studies outlined opportunities for improvement [11–15], elucidated the principal barriers to team effectiveness [16–20] and provided clear guidance on the characteristics of highly performing MDTs [17], the change management processes required to strengthen team performance have received little attention.

For the past 20 years, Western Sydney Local Health District (WSLHD) has progressively introduced MDTs into its tumour programs (e.g. thoracic oncology, melanoma). By 2017, they were well-accepted and attended. However, their performance in areas such as governance, meeting organisation and clinical decision-making, was variable [8]. In 2017, the WSLHD Cancer and Haematology Services (CHS) established a structured improvement initiative (TPSI) to optimise MDT function.

The aim of this study was to determine if TPSI could lead to sustained improvement in performance.

In 2019, the authors published a paper describing the tools used in TPSI and provided early results [21]. This paper reports on subsequent outcomes from the program after three annual surveys.

## Methods

### TPSI program

WSLHD has two university teaching hospitals each with cancer MDTs. WSLHD CHS supports 12 MDTs (colorectal, gynaecological-oncology, head and neck, hepatocellular, lymphoma, melanoma, neuro-oncology, sarcoma, upper gastro-intestinal, uro-oncology and two for lung cancer). Breast Cancer is managed through a separate entity and was not included in the study. 11 MDTs were based in a larger referral hospital (A) and one in a smaller hospital (B). Most of the MDTs based at hospital A had a district wide scope.

**Fig. 1:**
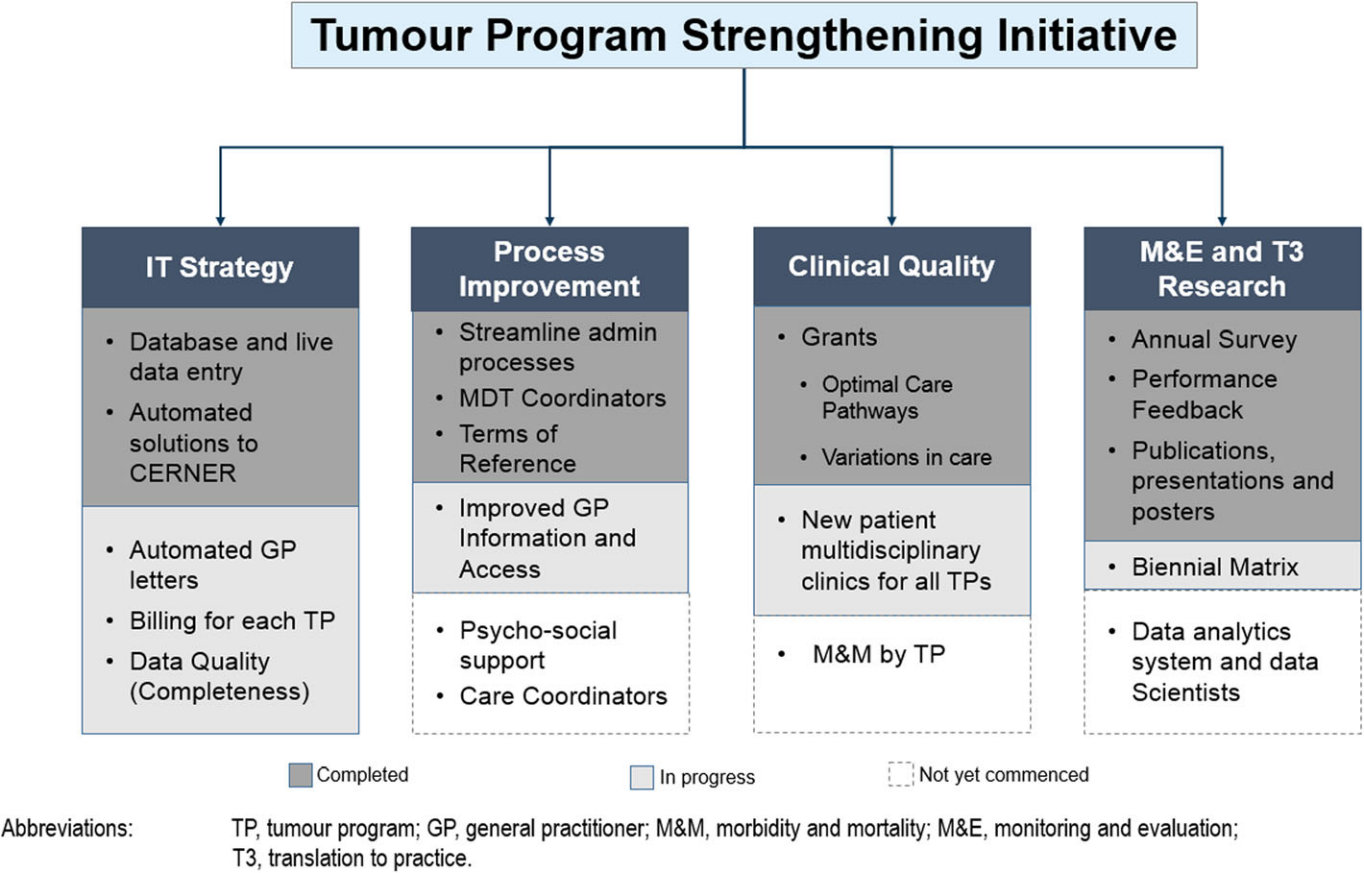
TPSI strategy with four components

The purpose of TPSI was ‘to improve the performance of all MDTs within CHS’. Its core components were information technology (IT), process improvement, clinical quality, and monitoring, evaluation and health services research (Fig. 1).

Early activities focussed on the IT strategy with particular emphasis on establishing an MDT database that was integrated into the Oncology Information System (OIS). This streamlined a variety of administrative processes, including referral and registration of patients, documentation of consensus in the meeting, and recording of attendance. Establishing TORs, with defined criteria for referral was also a key focus.

When opportunities arose, clinical quality projects were initiated with grant funding from the New South Wales (NSW) Cancer Institute. Through a mixture of team workshops, stakeholder interviews and audits, the authors investigated timeliness of care and compliance with identified indicators, identified key constraints to timely care and, where possible, implemented strategies to address issues found.

### MDT member survey

An MDT member survey was used to monitor the team’s progress over time and to evaluate the success of the TPSI strategy. The survey also served to inform team members of the attributes of highly performing teams and to identify areas in need of improvement. Each team agreed to complete a survey at the beginning of the program and annually thereafter. Completion of the MDT survey by the team members, after having been informed of the purpose of the survey, was considered to be informed consent.

In 2017, a 43-question on-line Survey Monkey questionnaire, adapted from one previously developed by Rankin et al. [13], was administered to assess members’ perceptions of their teams’ performance over multiple domains. Members participating in more than one MDT were asked to complete a survey for each one. The responses were analysed using the Survey Monkey data analysis software. Filters were used to isolate the results for each MDT. The authors analysed the responses for all 43 questions and compared the results for each MDT with the combined results for the district. A summary of the findings was presented to each team.

As a result of feedback from the 2017 survey, a revised and simplified version was repeated in 2018. Minor changes were also made in 2019. The format for Question 6, concerning whether decisions were sometimes ad hoc, was changed slightly and responses were expanded from yes/no to include a range from always to never. While it is understood that this could affect the validity of the result, it has been included because of the importance of the question. Questions from the structured survey instrument can be found in Additional File 1. Respondents can only choose one answer from the list of response options for each question.

Data was analysed for 17 of the survey questions that were asked in all three years to detect statistical differences in the proportion of positive responses between 2017 and 2019. The statistical software IBM SPSS Statistics 24 was used to analyse the data. Two-tailed tests with a significance level of 5% were used throughout. Because members of the same team tended to have responses that were more similar to each other than to responses from members of different teams, each participating team was considered a ‘cluster’. GEEs with a robust covariance matrix and exchangeable correlation structure were used to fit logit models and test for a difference between 2017 and 2019 in the proportion of ‘positive’ responses to each question adjusted for team clustering. The cluster-adjusted odds ratio (OR) of a ‘positive’ response in 2019 vs 2017 and their 95% confidence intervals (95% CI) were used to quantify the change. The associated cluster-adjusted *P* value is reported.

### Patient and public involvement

This study did not have public or patient involvement.

## Results

### TPSI program

Figure 1 outlines the program strategy and shows the activities that have commenced over the past two years. Eleven of 12 teams are now using the database with a consensus plan agreed and documented live in meetings. Eleven of 12 teams have formally endorsed TORs, which include criteria for referring patients to the MDT. Administrative processes have been automated and streamlined and support for team meetings has been considerably strengthened.

Cancer Institute funded projects demonstrated many opportunities for improvement and outlined recommendations, implementation of which is ongoing. Lung, colorectal and liver cancer projects have been completed. All found considerable delays in care at some stage of the process. In addition, the colorectal cancer project found contraints with respect to accessing colonoscopies and variations in adherence to Enhanced Recovery after Surgery (ERAS) protocols. The liver team also identified access issues for interventional radiology. The lung and liver teams identified constraints with meeting organisation. All the projects identified the need for dedicated care coordinators.

### MDT survey

#### Combined results from 12 MDTs

In 2017, 129 survey responses were received (57% response rate). one hundred eighteen members (52%) completed the survey in 2018 and 146 (65%) in 2019. Eighty-eight to 91% of all responses were from Hospital A and 9 to 12% from Hospital B. Over the three years, the range of responses from the different disciplines was: treating specialists (64 to 66%); nursing staff (10 to 15%); diagnostic specialists (9 to 14%); research staff (3%); allied health (2%); and others (2 to 5%).

Table 1 shows the OR of positive responses, 95% CI and *P* values comparing 2017 to 2019 for the whole service. Nine questions showed statistically significant improvements when compared to baseline results from 2017. These are a designated position for documentation, TORs, referral criteria, follow-up processes, documentation of consensus, treatment decision-making, use of clinical practice guidelines, informing patients, and discussion of patient preferences. Changes in positive responses were highly significant for five of these questions. The raw data for this analysis can be found in Additional File 2.

**Table 1:** Cluster (team) adjusted OR and 95% CI for ‘positive’ responses comparing 2017 to 2019 and associated *P* values for the 19 questions

Survey Questions		OR 2019 vs 2017^**a**^	95% CI for OR		***P*** value^**b**^
			lower	upper	
**Meeting Organisation**					
1	Is there a dedicated person/position to document meeting outcomes?	5.1	2.6	10.0	<0.001**
2	Does the MDM have a Terms of Reference or guideline to guide the conduct of the meetings?	6.6	3.1	14.2	<0.001**
3	Are there established criteria to determine which types of patients should be referred to the MDM?	3.3	2.1	5.2	<0.001**
4	Is there a follow-up process to check whether referrals are actually made?	2.7	1.0	7.0	0.046*
**Clinical Decision Making**					
5	Is consensus documented for each patient as a result of discussion in the meeting?	14.8	3.6	61.1	<0.001**
6	How often are treatment decisions based on an individual clinician's preference rather than endorsed guidelines or published literature? (Always or Usually)	0.2^d^	0.1	0.3	<0.001**
7	Does the MDM refer to International, National or State Clinical Practice Guidelines or Standard Treatment Protocols when making management decisions for cancer patients from your tumour stream?	1.8	1.3	2.5	0.001*
**Patient Considerations**					
8	How often are patients informed that they will be discussed in the MDM?	2.5	1.4	4.3	0.002*
9	Is there a formal process for raising patient preferences in the MDM discussions?	2.2	1.3	3.8	0.05*
10	How often are patient preferences discussed in the MDM?	1.1	0.9	14.5	0.357
11	How often are supportive care needs (e.g. social, financial, psychological, or others) of patients discussed in the MDM?	1.2	0.9	1.7	0.148
12	Do you routinely collect whether the patient has a psych-oncology screening?	1.2	0.3	4.5	0.779
**Quality Improvement and Research**					
13	How often are quality improvement activities discussed in, or reported to, the MDM?	0.9	0.4	1.8	0.685
14	Are internal audits conducted to confirm that treatment decisions match current best practice?	0.9	0.4	1.9	0.725
15	Do you routinely collect time from diagnosis to active treatment?	1.6	0.8	3.3	0.174
16	Do you routinely collect % of patients seen by the MDM prior to commencement of treatment?	1.6	0.7	3.3	0.253
**Education/Professional Development**					
17	How often are professional development activities made available for MDM members?	0.5	0.2	1.2	0.119

^*^ Statistically significant; ^**^ Statistically highly significant

^a^ Odds ratio adjusted for team clustering; ^b^ *P* value adjusted for team clustering; ^d^ The proportion of ‘sometimes, always or usually’ responses decreased significantly in 2019

**Fig. 2:**
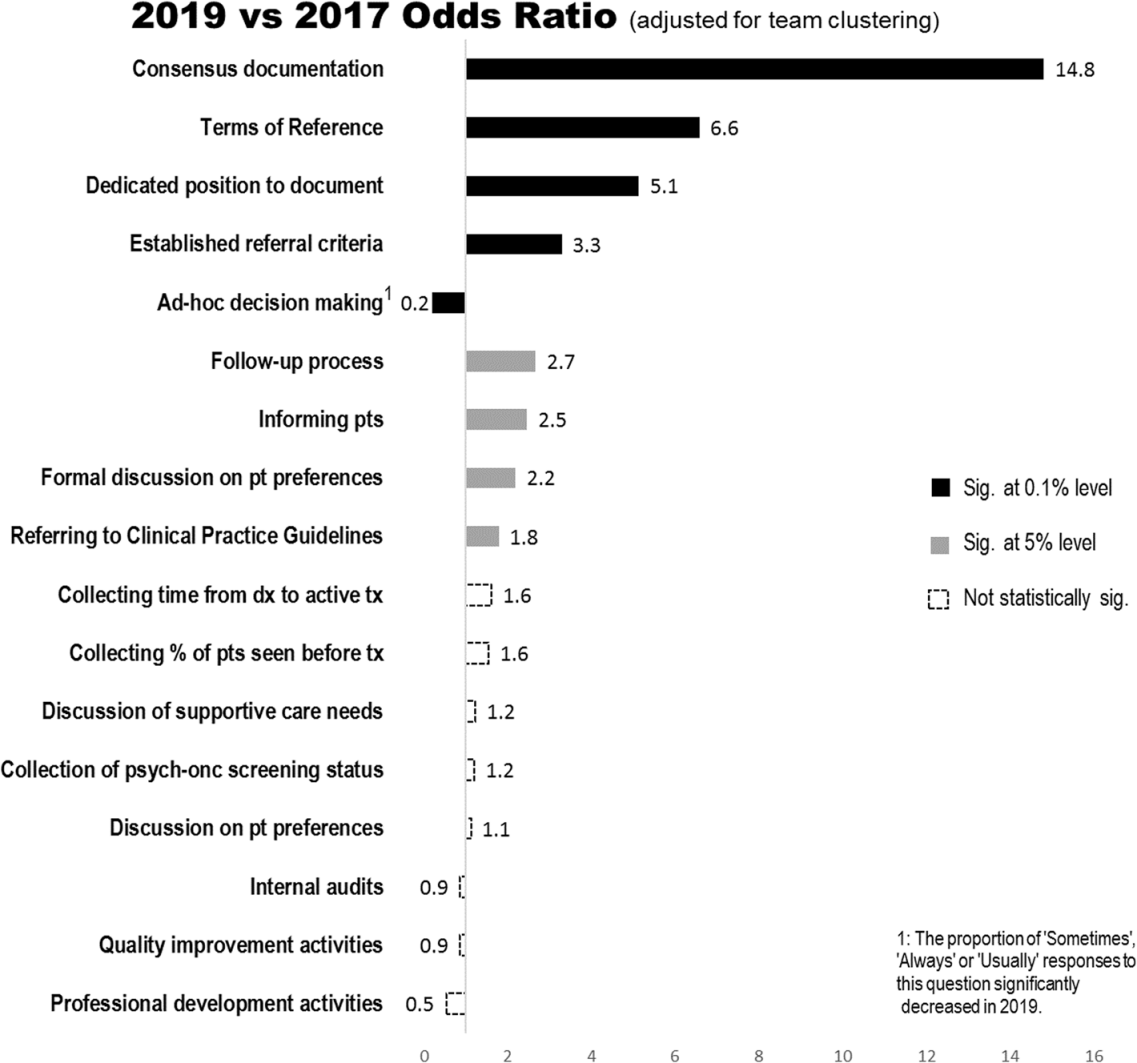
The relative odds ratios for 2019 to 2017

The cluster-adjusted ORs of positive responses to the 17 survey questions are shown diagrammatically in Fig. 2.

Questions 1 to 4 relating to operational matters all improved significantly with responses to questions relating to documentation [1], TORs [2] and criteria for referral [3] being highly significant. Q4, relating to follow-up processes, increased from 14 to 30% (OR = 2.7, 95% CI: 1.0–7.0, *P* = 0.046).

Questions 5 to 7, relating to clinical decision making, all showed significant improvement. Reported documentation of consensus for each patient as a result of discussion in the meeting increased from 83 to 99% (OR = 14.8, 95% CI: 3.9–61.1, *P* < 0.001*)*. 39% of members reported that treatment decisions were always or usually based on an individual clinician’s preference in 2017. This proportion decreased to 10% in the 2019 survey (OR = 0.2, 95% CI: 0.1–0.3, *P <* 0.001). The percentage of members responding that they referred to clinical practice guidelines increased from 63 to 77% over the two years (OR = 1.8, 95% CI: 1.3–2.5, *P* = 0.001).

Other results were less positive. While significant improvements were found in members’ responses about informing patients of the MDT discussion (OR = 2.5, 95% CI: 1.4–4.3, *P* = 0.002) and formally raising patient preferences during MDT meeting (OR = 2.2, 95% CI: 1.3–3.8, *P* = 0.05), questions about discussion of patient preferences and supportive care needs did not improve significantly. Very few members reported that they collected patient’s psycho-oncology screening status on a routine basis and this did not increase during the study period.

Questions relating to quality improvement and professional development did not show any significant improvement over the three years. The proportion of positive responses on professional development activities (question 17) increased slightly between 2017 (28%) and 2018 (30%) but decreased significantly between 2018 and 2019 (17%).

#### Team comparison

Table 2 compares the percentage of positive responses for each of the 12 teams. While the number of responses for each team was too small for a statistical analysis, there was a marked increase in scores (percentages of positive responses) over the three years for all teams.

**Table 2:** Comparison of percentages of positive responses to survey questions in 2017 and 2019, ranked by total score of each team in 2019

	Question	Year	WSLHD	Multidisciplinary teams											
				7	10	9	12	1	8	11	6	3	4	5	2
**Meeting Organisation**															
1	Is there a dedicated person/position to document meeting outcomes? (yes or no)	2017	**66**	90	50	90	77	100	45	40	93	38	30	60	60
		2019	**91**	100	100	100	91	94	80	75	100	100	92	73	75
2	Does the MDM have a Terms of Reference or guideline to guide the conduct of the meetings? (yes or no)	2017	**15**	30	43	10	8	36	9	0	0	25	0	20	0
		2019	**55**	81	82	54	64	71	38	50	43	40	25	43	13
3	Are there established criteria to determine which types of patients should be referred to the MDM? (yes or no)	2017	**26**	70	43	50	38	18	36	10	8	25	0	0	10
		2019	**55**	69	54	85	45	65	80	50	60	58	33	9	25
4	Is there a follow-up process to check whether referrals are actually made? (yes or no)	2017	**14**	30	57	30	8	0	18	0	0	0	0	10	0
		2019	**30**	69	54	62	45	18	10	8	20	8	8	18	25
**Clinical Decision Making**															
5	Is consensus documented for each patient as a result of discussion in the meeting? (always and usually)	2017	**78**	90	83	100	100	91	82	90	86	75	30	80	80
		2019	**99**	100	100	100	100	100	100	100	100	92	100	100	88
6	How often are treatment decisions based on an individual clinician's preference rather than endorsed guidelines or published literature? (rarely or never)	2017	**62**	60	42	80	62	91	64	50	79	62	60	30	50
		2019	**89**	100	85	91	82	100	100	83	80	92	92	82	75
7	Does the MDM refer to International, National or State Clinical Practice Guidelines or Standard Treatment Protocols when making management decisions for cancer patients from your tumour stream? (always and usually)	2017	**63**	80	64	80	69	100	82	30	58	63	50	30	40
		2019	**76**	100	69	85	73	88	90	67	70	92	67	45	38
**Patient Considerations**															
8	How often are patients informed that they will be discussed in the MDM? (always and usually)	2017	**55**	40	58	80	38	64	73	70	64	38	40	60	20
		2019	**75**	63	85	92	91	72	80	100	50	50	92	73	38
9	Is there a formal process for raising patient preferences in the MDM discussions? (yes or no)	2017	**16**	60	8	0	15	9	27	20	14	25	0	0	30
		2019	**32**	63	31	46	36	22	40	0	50	33	17	27	0
10	How often are patient preferences discussed in the MDM? (always and usually)	2017	**59**	80	75	60	31	64	82	60	79	25	50	40	30
		2019	**60**	69	77	67	36	78	80	75	60	42	42	45	38
11	How often are supportive care needs (e.g. social, financial, psychological, or others) of patients discussed in the MDM? (always and usually)	2017	**26**	70	17	20	38	30	27	30	50	13	20	10	0
		2019	**33**	69	54	25	27	39	30	42	30	17	17	9	25
12	Do you routinely collect whether the patient has a psych-oncology screening? (yes or no)	2017	**2**	0	0	0	8	0	9	0	7	0	0	0	0
		2019	**8**	13	15	0	0	0	0	0	0	0	0	0	0
**Quality Improvement and Research**															
13	How often are quality improvement activities discussed in, or reported to, the MDM? (at least quarterly)	2017	**18**	20	0	50	31	55	9	0	29	13	0	10	0
		2019	**16**	38	15	8	36	17	20	17	10	8	8	0	13
14	Are internal audits conducted to confirm that treatment decisions match current best practice? (yes or no)	2017	**7**	20	14	0	8	9	9	0	0	0	10	10	0
		2019	**6**	25	8	0	18	0	0	0	0	0	17	0	0
15	Do you routinely collect time from diagnosis to active treatment? (yes or no)	2017	**10**	20	14	33	31	0	0	0	0	25	0	0	0
		2019	**16**	25	15	25	27	18	10	33	0	17	8	0	0
16	Do you routinely collect % of patients seen by the MDM prior to commencement of treatment? (yes or no)	2017	**10**	20	0	20	15	0	9	0	29	13	0	0	0
		2019	**14**	25	15	8	9	6	20	25	20	8	8	9	13
**Professional Development**															
17	How often are professional development activities made available for MDM members? (at least quarterly)	2017	**28**	10	0	60	31	73	18	60	29	0	40	10	0
		2019	**17**	38	31	8	36	22	0	25	0	8	0	0	25
**Total Score**		**2017**	**549**	**790**	**568**	**763**	**608**	**740**	**599**	**460**	**625**	**440**	**330**	**370**	**320**
		**2019**	**868**	**1197**	**990**	**929**	**907**	**957**	**938**	**816**	**774**	**699**	**684**	**615**	**541**

For the 17 questions, where 2017 and 2019 can be compared, the average score for the 12 teams increased from 549 to 868 with the highest performing team [7] increasing from 790 to 1197 and the poorest performing team [2] increasing from 320 to 541. The maximum possible score is 17 X 100% or 1700.

## Discussion

### Statement of principle findings

This study shows that a structured multipronged approach to strengthening MDT performance across a whole service can lead to a significant increase in team performance as assessed by team members. An annual MDT member survey combined with a program of support engaged MDT members, provided a framework for strengthening performance and ultimately led to significant improvement in many parameters.

The highly significant improvement in Q1 to 3 and significant improvement in Q4, all relating to operation issues was gratifying as this was the early focus of the study.

The improvement in Q5 may be explained by the inclusion of a field for consensus documentation with live data entry. The reason for the positive findings for questions 6 and 7 could be the increased awareness of their importance because of the survey or may reflect more generally the increased engagement with MDT team members and the focus being placed on MDT performance [22].

We cannot explain the significant improvement in the number of members responding that there was a formal process for raising patient preferences (Q9) (OR = 2.2, 95% CI: 1.3–3.8, *P* = 0.05) particularly as responses to other questions relating to patient wellbeing (Q11 to 13) showed no significant change. We believe the significant increase in those responding that they routinely inform patients (Q8) was a direct result of having the question in the survey and an understanding that it was required for billing purposes.

Questions relating to quality improvement and research did not show significant change. The authors were disappointed that no teams initiated audits and will continue to encourage this. Audits are constrained by the available IT systems and personnel to assist with data collection.

The significant reduction in members responding that they had professional development activities within MDT meetings at least quarterly is disappointing. The 2017 and 2018 surveys included a preceding question that outlined the activities that were considered to be professional development. This question was removed in 2019 to simplify the survey. Thus, previous respondents may have forgotten, and new members may not have understood, the terminology.

The comparison between MDTs provided insights on how each team performed compared to others in the same institution. Teams that scored highly worked hard to maintain their position. While movements in the rankings were generally one or two places, they were keenly followed by team members. The results of the survey reinforced the wider goal of the TPSI program which is not simply to monitor performance but to inspire major and sustainable organisational change over time.

### Strengths and limitations

The strength of this study is that it demonstrates a simple method to measure MDT members’ perceptions of their performance and engage clinicians to change practices. Self-assessment ensured both that the responses reflected the opinion of most team members and that it was not overly burdensome for clinicians or for management. A high level of engagement from team members was indicated by the consistently high completion rate for the survey and the significant improvement for many questions.

The limitation of this study is that the MDT survey only investigates members’ perceived performance of MDTs and may be subject to respondent bias. The results may not be generalisable to other regions as MDT members who participated in this study were recruited from the same Local Health District in Sydney.

### Interpretation within the context of the wider literature

Many articles describe the functioning of MDTs, [13, 14] the characteristics of well-performing teams, [4–6, 23] or examples of improved performance in individual teams led by motivated individuals [1, 24]. Lamb outlined several strategies to improve the efficiency and utility of multidisciplinary teams [25].

A few groups have outlined tools – both electronic and paper based – to strengthen MDTs. Soukup et al. provide an excellent summary of the recent literature [20]. The Western and Central Melbourne integrated cancer service provided paper-based tools to help with organising the MDT [23]. Patkar et al. have demonstrated benefits from a decision support system, [26] and Nouraei et al. have shown that a database aimed at ensuring that patients were presented after all their relevant investigation had been completed, demonstrably improved efficiency [27]. Several teams have developed and validated tools for teams to measure their performance [23, 28, 29].

This study filled a gap by combining a structured improvement program with an annual survey to measure the performance of each team. In addition, it targetted all tumour streams within the LHD, regardless of their level of maturity.

### Implications for policy, practice and research

By developing a comprehensive evidence-based strategy to systematically improve all aspects of MDTs, this study provides a methodology for health services attempting to strengthen MDT performance.

It provides management with insights about areas that need improving across the board and allows it to identify poorly performing teams so they can be supported more intensively.

The survey can also be used to monitor how other research interventions affect the overall performance of MDTs.

## Conclusions and future directions

This study demonstrates that a structured program to strengthen MDT performance can produce significant behaviour change. The annual MDT member survey not only monitors performance but also provides a means for teams to identify their own strengths and weaknesses. In addition, it allows management to review all MDTs against standardised criteria and to determine further support required.

The significance of this initiative is that overall program improvement reflects the strengthening of the weakest teams as well as further improvement in already highly performing MDTs.

While the foundations have been laid, the program will be continually adapted. A key focus will be quality improvement. New projects are being initiated for bladder cancer and to extend the lung project. The authors will continue to support teams wishing to apply for grants in the future.

The authors will continue to advocate for resources to address those recommendations that cannot be implemented without additional funding. The appointment of care coordinators for each team would support psych-oncology screening and focus attention on patient wellbeing.

An increased emphasis on collection of performance indicators and data quality is envisaged for the future with a data analytics package linked with the OIS scheduled for 2021. This will allow the establishment of real-time dashboards for quality indicators. Resources for data collection and analysis will be important if this is to be successful.

## Supplementary Information

**Additional file 1.** MDT Survey. Structured survey instrument used in this study.

**Additional file 2.** 2017–2019 Survey Data. 2017 to 2019 MDT member survey data.

## Data Availability

All data generated or analysed during this study are included in this published article and its supplementary information files.

## Abbreviations

CHS: Cancer and Haematology Services
CI: Confidence Interval
GEE: Generalised Estimating Equation
MDT: Multidisciplinary Team
NSW: New South Wales
OCP: Optimal Care Pathway
OIS: Oncology Information System
OR: Odds Ratio
QI: Quality Improvement
TORs: Terms of Reference
TPSI: Tumour Program Strengthening Initiative
WSLHD: Western Sydney Local Health District
