# Supplementary material for: Improving Cancer MDT performance in Western Sydney – three years’ experience
Source: BMC Health Serv Res. 2021 Mar 6;21:203. doi: 10.1186/s12913-021-06203-y (PMC7937192; doi:10.1186/s12913-021-06203-y)
Supplement: Supplementary file 1 — Additional file 1. MDT Survey. Structured survey instrument used in this study. [file 12913_2021_6203_MOESM1_ESM.pdf]

2019 Annual Survey

Don't forget to click the **Done** button after finishing the survey. Your responses will be lost otherwise.

* 1. Please provide the MDM you are answering this survey about.

- ◯ Colorectal
- ◯ Gynae-oncology
- ◯ HCC
- ◯ Head & Neck
- ◯ Joint Lymphoma
- ◯ Leukaemia
- ◯ Lung Blacktown
- ◯ Lung Westmead
- ◯ Melanoma
- ◯ Neuro-oncology
- ◯ Sarcoma
- ◯ UGIT
- ◯ Urology

* 2. Please select your role in the MDM.

- ◯ Treating Specialist (Medonc, Radonc, Haem, Respiratory, Surgery, Pall Care)
- ◯ Diagnostic Specialist (Radiology/Pathology/Nuclear Med)
- ◯ Nursing
- ◯ Admin Support
- ◯ Allied Health
- ◯ Clinical Trials/Research/Biobank
- ◯ Data Analysis
- ◯ Other (please specify)

* 3. Please answer the following questions relating to the running of the MDM.

	Yes	No	Unsure
Is there a dedicated person/position to document meeting outcomes?	◯	◯	◯
Are there established criteria to determine which types of patients should be referred to the MDM ?	◯	◯	◯
Does the MDM have a policy of registering all patients - even those not being referred to the MDM?	◯	◯	◯
Is there a follow-up process to check whether referrals are actually made?	◯	◯	◯
Does the MDM have a Terms of Reference or guideline to guide the conduct of the meetings?	◯	◯	◯

4. Is consensus documented for each patient as a result of discussion in the meeting?

- ◯ Always
- ◯ Usually
- ◯ Sometimes
- ◯ Rarely
- ◯ Never

5. Is there a formal process for prioritising patients for referral to the MDM?

- ◯ Yes - prioritisation has been decided
- ◯ Yes - All cases are referred to the MDM
- ◯ No
- ◯ Unsure

* 6. How often are treatment decisions based on an individual clinician's preference rather than endorsed guidelines or published literature?

- ☐ Always
- ☐ Usually
- ☐ Sometimes
- ☐ Rarely
- ☐ Never
- ☐ Unsure
- ☐ Other (please specify)

* 7. Does the MDM refer to International, National or State Clinical Practice Guidelines or Standard Treatment Protocols when making management decisions for cancer patients from your tumour stream?

- Always
- Usually
- Sometimes
- Rarely
- Never
- Unsure
- Other (please specify)

* 8. Please answer the following questions relating to decision making.

	Yes	No	Unsure
Have any Clinical Practice Guidelines or Treatment Protocols been formally endorsed by the Tumour Program?			
Are internal audits conducted to confirm that treatment decisions match current best practice?			
Are internal audits conducted to check if treatment decisions match MDM recommendations?			
Is there a formal process for raising patient preferences in the MDM discussion?			

NSW GOVERNMENT Health Western Sydney Local Health District

2019 Annual Survey

Don't forget to click the **Done** button after finishing the survey. Your responses will be lost otherwise.

* 9. Please answer the following questions regarding patient needs.

	Always	Usually	Sometimes	Rarely	Never	Unsure
How often are patients informed that they will be discussed in the MDM?	◯	◯	◯	◯	◯	◯
How often are supportive care needs (e.g. social, financial, psychological, or others) of patients discussed in the MDM?	◯	◯	◯	◯	◯	◯
How often are patient preferences discussed in the MDM?	◯	◯	◯	◯	◯	◯
How often do MDM discussions result in referrals to psycho-oncology services?	◯	◯	◯	◯	◯	◯
How often do MDM discussions result in referrals to other allied health services?	◯	◯	◯	◯	◯	◯
How often is clinical trial eligibility discussed?	◯	◯	◯	◯	◯	◯

If you wish to add a comment please indicate below.

* 10. Please answer the following questions about Quality Improvement and Professional Development activities.

	At least monthly	At least quarterly	On an ad hoc basis when available	Never - does not occur	Never - occurs elsewhere	Not sure
How often are quality improvement activities discussed in, or reported to, the MDM?	◯	◯	◯	◯	◯	◯
How often are professional development activities made available for MDM members?	◯	◯	◯	◯	◯	◯

If you wish to add a comment please indicate below.

* 11. Do you collect the following data routinely?

	Yes	No	Unsure
Time from diagnosis to active treatment	◯	◯	◯
% of patients with the condition routinely seen by MDM	◯	◯	◯
% of patients who are seen by the MDM prior to commencement of treatment	◯	◯	◯
Whether the patient had validated psycho-oncology screening?	◯	◯	◯

* 12. Do you collect the following information routinely?

	Yes	No	Unsure
Diagnosis	◯	◯	◯
Site	◯	◯	◯
Stage (TNM/other)	◯	◯	◯
ECOG status	◯	◯	◯
Treatment Intent	◯	◯	◯
Patient Status (New Patient/Follow Up)	◯	◯	◯
Presenting Symptoms	◯	◯	◯
Referrals	◯	◯	◯

MDMs are now using the Oncology Information System to collect the avobe information, so this question was excluded from statistical analysis.

* 13. How often does at least one member of the following teams attend the MDM?

	Always	Usually	Sometimes	Rarely	Never	Unsure	Not Applicable
Palliative and Supportive Care	◯	◯	◯	◯	◯	◯	◯
Radiology	◯	◯	◯	◯	◯	◯	◯
Psyche Oncology	◯	◯	◯	◯	◯	◯	◯
Social Work	◯	◯	◯	◯	◯	◯	◯
Clinical Trials	◯	◯	◯	◯	◯	◯	◯
Admin Officer	◯	◯	◯	◯	◯	◯	◯
Data Manager	◯	◯	◯	◯	◯	◯	◯

MDMs are now using the Oncology Information System to record attendance, so this question was excluded from statistical analysis.

NSW GOVERNMENT Health Western Sydney Local Health District

2019 Annual Survey

Don't forget to click the **Done** button after finishing the survey. Your

responses will be lost otherwise.

14. Do you have any additional comments about MDM’s or your facility?
