# Supplementary material for: Improving Cancer MDT performance in Western Sydney – three years’ experience
Source: BMC Health Serv Res. 2021 Mar 6;21:203. doi: 10.1186/s12913-021-06203-y (PMC7937192; doi:10.1186/s12913-021-06203-y)
Supplement: Supplementary file 2 — Additional file 2. 2017–2019 Survey Data. 2017 to 2019 MDT member survey data. [file 12913_2021_6203_MOESM2_ESM.pdf]

# 2017, 2018, 2019 Survey Results

| | 2017 | 2018 | 2019 |
|---|---|---|---|
| **Meeting Organisation** | | | |
| 1. Is there a dedicated person/position to document meeting outcomes? ('Yes') | | | |
| **n** | 85 | 92 | 132 |
| **N** | 129 | 115 | 145 |
| **%** | 65.9% | 80.0% | 91.0% |
| 2. Does the MDM have a Terms of Reference or guideline to guide the conduct of the meetings? ('Yes') | | | |
| **n** | 20 | 54 | 69 |
| **N** | 129 | 117 | 125 |
| **%** | 15.5% | 46.2% | 55.2% |
| 3. Are there established criteria to determine which types of patients should be referred to the MDM? ('Yes') | | | |
| **n** | 34 | 62 | 80 |
| **N** | 129 | 117 | 146 |
| **%** | 26.4% | 53.0% | 54.8% |
| 4. Is there a follow-up process to check whether referrals are actually made? ('Yes') | | | |
| **n** | 18 | 22 | 44 |
| **N** | 129 | 117 | 146 |
| **%** | 14.0% | 18.8% | 30.1% |
| **Clnicial Decision Making** | | | |
| 5. Is consensus documented for each patient as a result of discussion in the meeting? ('Always' and 'Usually') | | | |
| **n** | 107 | 108 | 144 |
| **N** | 129 | 115 | 146 |
| **%** | 82.9% | 93.9% | 98.6% |
| 6. How often are treatment decisions based on an individual clinician's preference rather than endorsed guidelines or published literature? ('Always' or 'Usually') | | | |
| **n** | 50 | 41 | 15 |
| **N** | 129 | 115 | 146 |
| **%** | 38.8% | 35.7% | 10.3% |
| 7. Does the MDM refer to International, National or State Clinical Practice Guidelines or Standard Treatment Protocols when making management decisions for cancer patients from your tumour stream? ('Always' and 'Usually') | | | |
| **n** | 81 | 72 | 111 |
| **N** | 129 | 117 | 145 |
| **%** | 62.8% | 61.5% | 76.6% |
| **Patient Considerations** | | | |
| 8. How often are patients informed that they will be discussed in the MDM? ('Always' and 'Usually') | | | |
| **n** | 70 | 89 | 108 |
| **N** | 129 | 115 | 145 |

n: Number of 'positive' responses
N: Total number of responses
%: Percentage of 'positive' responses

# 2017, 2018, 2019 Survey Results

| | | | |
|---|---|---|---|
| **%** | 54.3% | 77.4% | 74.5% |
| 9. Is there a formal process for raising patient preferences in the MDM discussions? ('Yes') | | | |
| **n** | 22 | 19 | 46 |
| **N** | 129 | 115 | 146 |
| **%** | 17.1% | 16.5% | 31.5% |
| 10. How often are patient preferences discussed in the MDM? ('Always' and 'Usually') | | | |
| **n** | 74 | Not included | 88 |
| **N** | 129 | | 145 |
| **%** | 57.4% | | 60.7% |
| 11. How often are supportive care needs (e.g. social, financial, psychological, or others) of patients discussed in the MDM? ('Always' and 'Usually') | | | |
| **n** | 36 | 34 | 49 |
| **N** | 128 | 115 | 145 |
| **%** | 28.1% | 29.6% | 33.8% |
| 12. Do you routinely collect whether the patient has a psych-oncology screening? ('Yes') | | | |
| **n** | 3 | 1 | 4 |
| **N** | 129 | 115 | 145 |
| **%** | 2.3% | 0.9% | 2.8% |
| **Quality Improvement and Research** | | | |
| 13. How often are quality improvement activities discussed in, or reported to, the MDM? ('At least quarterly') | | | |
| **n** | 24 | 16 | 24 |
| **N** | 129 | 115 | 145 |
| **%** | 18.6% | 13.9% | 16.6% |
| 14. Are internal audits conducted to confirm that treatment decisions match current best practice? ('Yes') | | | |
| **n** | 9 | 9 | 9 |
| **N** | 129 | 117 | 146 |
| **%** | 7.0% | 7.7% | 6.2% |
| 15. Do you routinely collect time from diagnosis to active treatment? ('Yes') | | | |
| **n** | 13 | 22 | 23 |
| **N** | 128 | 117 | 145 |
| **%** | 10.2% | 18.8% | 15.9% |
| 16. Do you routinely collect % of patients seen by the MDM prior to commencement of treatment? ('Yes') | | | |
| **n** | 12 | 14 | 20 |
| **N** | 128 | 115 | 145 |
| **%** | 9.4% | 12.2% | 13.8% |
| **Education/Professional Development** | | | |
| 17. How often are professional development activities made available for MDM members? ('Always' and 'Usually') | | | |
| **n** | 36 | 34 | 25 |
| **N** | 129 | 115 | 145 |
| **%** | 27.9% | 29.6% | 17.2% |

n: Number of 'positive' responses
N: Total number of responses
%: Percentage of 'positive' responses
